# Cost-utility analysis of caspofungin and fluconazole for primary treatment of invasive candidiasis and candidemia in Ethiopia

**DOI:** 10.1186/s12913-022-08662-3

**Published:** 2022-10-29

**Authors:** Gebremedhin Beedemariam Gebretekle, Atalay Mulu Fentie, Girma Tekle Gebremariam, Eskinder Eshetu Ali, Daniel Asfaw Erku, Tinsae Alemayehu, Workeabeba Abebe, Beate Sander

**Affiliations:** 1 Institute of Health Policy, Management and Evaluation, University of Toronto, Toronto, ON Canada; 2 Toronto Health Economics and Technology Assessment (THETA) Collaborative, University Health Network, Toronto General Hospital, Eaton Building, 10th Floor, Room 205 200 Elizabeth Street, M5G 2C4 Toronto, ON Canada; 3 School of Pharmacy, Addis Ababa University, Addis Ababa, Ethiopia; 4 University Centre for Applied Health Economics, School of Medicine & Menzies Health Institute Queensland, Griffith University, Griffith, QLD Australia; 5 Department of Pediatrics and Child Health, St. Paul’s Hospital and Millennium Medical College, Addis Ababa, Ethiopia; 6 American Medical Center, Specialty Center for Infectious Diseases and Travel Medicine, Addis Ababa, Ethiopia; 7 Department of Pediatrics and Child Health, School of Medicine, Addis Ababa University, Addis Ababa, Ethiopia; 8 ICES, Toronto, ON Canada; 9 Public Health Ontario, Toronto, ON Canada; 10 Centre for Research and Engagement in Assessment of Health Technology, Addis Ababa, Ethiopia

**Keywords:** Candidemia, Caspofungin, Echinocandin, Fluconazole, Invasive candidiasis, Cost-effectiveness, Ethiopia

## Abstract

**Background:**

Invasive candidiasis and/or candidemia (IC/C) is a common fungal infection leading to significant health and economic losses worldwide. Caspofungin was shown to be more effective than fluconazole in treating inpatients with IC/C. However, cost-effectiveness of caspofungin for treating IC/C in Ethiopia remains unknown. We aimed to assess the cost-utility of caspofungin compared to fluconazole-initiated therapies as primary treatment of IC/C in Ethiopia.

**Methods:**

A Markov cohort model was developed to compare the cost-utility of caspofungin versus fluconazole antifungal agents as first-line treatment for adult inpatients with IC/C from the Ethiopian health system perspective. Treatment outcome was categorized as either a clinical success or failure, with clinical failure being switched to a different antifungal medication. Liposomal amphotericin B (L-AmB) was used as a rescue agent for patients who had failed caspofungin treatment, while caspofungin or L-AmB were used for patients who had failed fluconazole treatment. Primary outcomes were expected quality-adjusted life years (QALYs), costs (US$2021), and the incremental cost-utility ratio (ICUR). These QALYs and costs were discounted at 3% annually. Cost data was obtained from Addis Ababa hospitals while locally unavailable data were derived from the literature. Cost-effectiveness was assessed against the recommended threshold of 50% of Ethiopia’s gross domestic product/capita (i.e.,US$476). Deterministic and probabilistic sensitivity analyses were conducted to assess the robustness of the findings.

**Results:**

In the base-case analysis, treatment of IC/C with caspofungin as first-line treatment resulted in better health outcomes (12.86 QALYs) but higher costs (US$7,714) compared to fluconazole-initiated treatment followed by caspofungin (12.30 QALYs; US$3,217) or L-AmB (10.92 QALYs; US$2,781) as second-line treatment. Caspofungin as primary treatment for IC/C was not cost-effective when compared to fluconazole-initiated therapies. Fluconazole-initiated treatment followed by caspofungin was cost-effective for the treatment of IC/C compared to fluconazole with L-AmB as second-line treatment, at US$316/QALY gained. Our findings were sensitive to medication costs, drug effectiveness, infection recurrence, and infection-related mortality rates. At a cost-effectiveness threshold of US$476/QALY, treating IC/C patient with fluconazole-initiated treatment followed by caspofungin was more likely to be cost-effective in 67.2% of simulations.

**Conclusion:**

Our study showed that the use of caspofungin as primary treatment for IC/C in Ethiopia was not cost-effective when compared with fluconazole-initiated treatment alternatives. The findings supported the use of fluconazole-initiated therapy with caspofungin as a second-line treatment for patients with IC/C in Ethiopia.

## Introduction

Invasive candidiasis and/or candidemia (IC/C) is an increasingly common fungal infection worldwide and has been associated with high rates of mortality, hospitalization, and healthcare cost [1–3]. Its incidence is estimated to be 3–5 per 100,000 persons in the general population and 1–2% of all medical and surgical ICU admissions [3]. IC/C attributed mortality rates vary widely, ranging from 10 to 47% [3], and patients’ hospital stay is 22–34 days longer compared to those with non-invasive candidiasis [1]. The total healthcare cost per patient with IC/C infection was estimated to range from US$ 48,487 to $157,574, with an average cost of $10,216 to US$ 37,715 per hospitalization [2]. In a study conducted in the US, candidemia was associated with a 14.5% increase in mortality, 10.1-days increase in hospital stay, and attributable costs of∼US$40,000 per patient [4].

Data on IC/C from low-and middle-income countries (LMICs) are scarce, but the few available studies demonstrate a high incidence and very high mortality rate [2, 5]. In Ethiopia, Fungi are estimated to infect approximately 8% of the population each year, and the number of persons at risk of IC/C is increasing, owing to the expansion of intensive care units (ICU), and high prevalence of HIV/AIDs, malignancies, chronic diseases and other risk factors [6]. The paucity of evidence on the burden of fungal infections, particularly IC/C, is challenging for healthcare planning [6, 7]. In 2017, a comprehensive assessment of the literature on fungal infection in Ethiopia reported that no study on nosocomial fungal infections had been published [7]. A recent study estimated an annual incidence of 5,300 candidemia cases and over 3,600 associated death, assuming a 5 per 100,000 person-year annual incidence and that up to 5% of all hospital beds serve as ICU beds [6].

The burden of IC/C, however, can be reduced by timely treatments with antifungal medications [8, 9]. International guidelines recommend using echinocandins as first-line treatment over azole or polyene antifungals, due to their enhanced clinical outcomes and safety profile [10–12]. Ethiopia has no local treatment guideline for IC/C, making its management a difficult task for clinicians. Furthermore, there is a lack of local evidence on fungal infection epidemiology and insufficient diagnostic options, with hospitals lacking basic yeast diagnostics as well as antifungal susceptibility testing procedures. As a result, antifungal treatment for IC/C has remained empiric [6, 13].

Caspofungin and fluconazole are the most commonly used antifungals for first-line therapy of IC/C, and they are used interchangeably due to the improved therapeutic benefit and affordability, respectively. Likewise, second-line antifungal agents are used mostly interchangeably. We therefore aimed to evaluate the cost-utility of using caspofungin as empiric first-line therapy followed by Liposomal amphotericin B (L-AmB) compared to fluconazole-initiated empiric therapy followed by caspofungin or L-AmB for the treatment of hospitalized patients with IC/C in Ethiopia, a low-resource setting country.

## Methods

A Markov cohort model was constructed to assess the cost-utility of using caspofungin or fluconazole antifungal agents as empiric first-line therapy, i.e., in the absence of microbiology workup or while awaiting for culture and susceptibility data, for Ethiopian adult inpatients with IC/C. This study was carried out from the Ethiopian health system perspective over a lifetime horizon. Consistent with the health system perspective, we included direct medical costs such as drug acquisition cost, hospitalization costs, cost of diagnosis and monitoring tests. Primary outcomes were expected life years (LYs), quality-adjusted life years (QALYs), costs (US$ 2021), and the incremental cost-utility ratio (ICUR) expressed in US$ per QALY gained. The ICUR was calculated as the difference in cost between the strategies divided by the difference in effectiveness (QALYs). QALYs were determined by multiplying the years lived in a given health state with the utility weights of that state [14]. Costs and QALYs were discounted at an annual rate of 3%, as recommended for LMICs [15]. Ethiopia has not established a cost-effectiveness threshold. The World Health Organization (WHO) recommends a cost-effectiveness threshold of 1–3 times GDP/capita [15]. However, in recent years, the use of this threshold has been widely questioned for a lack of scientific underpinnings to guide resource allocation decisions [16, 17]. Hence, we compared our ICUR values against the recently recommended threshold of 50% of a country’s gross domestic product (GDP)/capita for LMICs [17]. Ethiopia’s GDP per capita at the time of the study was US$952 [18]. The study was designed, conducted, and reported following the Consolidated Health Economic Evaluation Reporting Standards (CHEERS) statement [19].

### Treatment strategies

We compare three treatment strategies: ***(1)****Caspofungin treatment followed by L-AmB (CASPO -> L-AmB)*: patient received intravenous caspofungin (loading dose 70 mg on day 1, then 50 mg daily maintenance dose for 14 days) and those who had experienced treatment failure were switched to an additional 14 days of L-AmB (3 mg/kg per day for an average weight of 70 kg); **(2)***Fluconazole treatment followed by caspofungin (FLU -> CASPO -> L-AmB)*: patient received fluconazole oral (800-mg loading dose, then 400 mg daily for 14 days) and those who had failed to respond to fluconazole were switched to caspofungin, with L-AmB being used as a rescue agent if infection persisted; **(3)***Fluconazole treatment followed by liposomal amphotericin B (L-AmB) (FLU -> L-AmB)*: patient took fluconazole oral (800-mg loading dose and 400 mg daily for 14 days thereafter) and if this treatment failed, L-AmB was used as the second-line therapy. In accordance with current practice in Ethiopia, we assumed that fluconazole and caspofungin would be prescribed for 14 days on average, regardless of their use as first- or second-line therapy. We consider the same treatment duration for L-AmB therapy. To evaluate each treatment separately, we assumed that patients who had failed therapy and/or those who had a recurrence would be managed with the same treatment as used for the previous episode. We assumed that patients were hospitalized throughout the treatment period and no patients had their medication dose titrated.

### Model structure

**Fig. 1:**
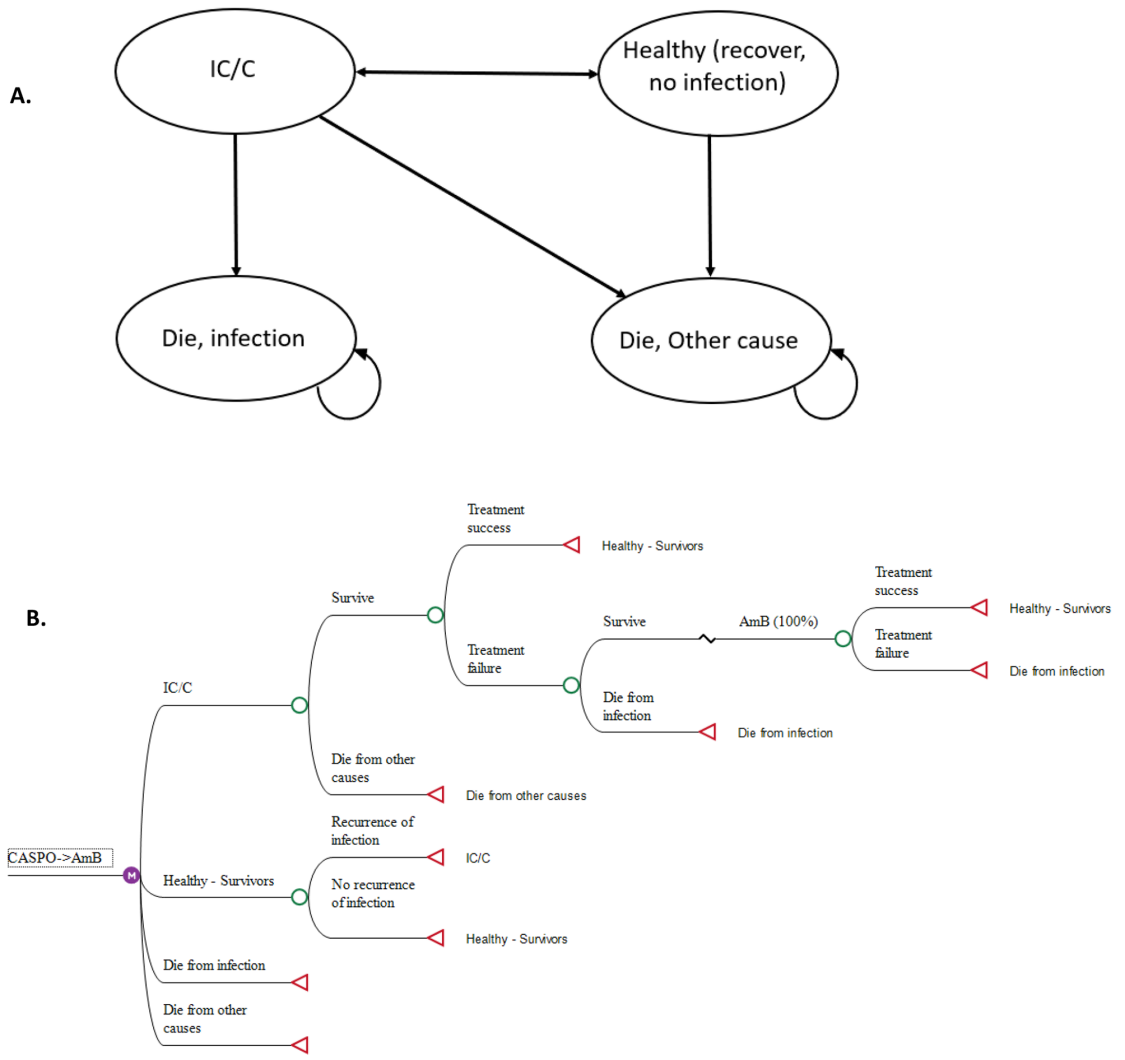
**A/B.** Schematic presentation of the model structure. **(A)** Patients spent each annual cycle in one of the health states: Oval represents health states while the arrows imply possible transitions of patients among different health states. **(B)** Illustrative model structure of the CASPO->L-AmB treatment strategy for IC/C patients. During each cycle, patients treated with any of the antifungal drugs could experience treatment success or failure. Those who had failed the first-line therapy could die due to the infection or survive and later would be switched to the next alternative therapy. Patients were also at risk of death due to causes unrelated to the infection. CASPO: Caspofungin; L-AmB: Liposomal amphotericin B

A Markov cohort model was constructed based on current clinical practice and treatment outcomes of hospitalized IC/C patients receiving different types of antifungal therapy in Ethiopia. We built the model using TreeAge Software (TreeAge Software, Inc., Williamstown, MA). Figure 1 shows a simplified illustration of the model structure. A patient in hospital with IC/C could die from infection or be cured and transition to a healthy state, which is defined as the complete resolution of the infection (i.e. clinical and microbiological success) with no need for additional systemic antifungal therapy [20]. Patients who were first treated and cured could either stay healthy or develop IC/C again. If the first-line treatment failed, patients would be switched to second-line antifungal treatment (Fig. 1B).

All patients could die from causes unrelated to IC/C. The simulated population reflects the Ethiopian inpatient with a mean age of 39 years (informed by hospital data). All patients were followed in a 3-months time step (cycle length) over their life expectancy.

### Parameter inputs

Model inputs including probabilities, utilities, and costs are reported in Table 1.

#### Probabilities

In the absence of local data, the literature was used to inform health state transitions. A meta-analysis by Millis et al. (2009) reported that caspofungin is superior, with favorable treatment response in 76.1% of patients, as compared to 63% for fluconazole and 72.98% for L-AmB [21]. The attributable mortality associated with IC/C was 28.44% in patients who received fluconazole and 33.83% with caspofungin. Recurrent candidemia was found in 4.4% of patients [22] and we assumed the same infection recurrence rate for all treatment strategies. Life expectancy data from the WHO Global Health Observatory for Ethiopia was used to populate age-specific mortality unrelated to IC/C [23].

#### Utilities

We derived utilities from the literature because local utility values for these patient populations were not available. Because Ethiopia’s general population mean utility is comparable to that of high-income nations [24], we used utilities from Western countries. The utility score for patients with IC/C (0.72) was extracted from the catalogue of preference scores 1997–2018 from the Cost-Effectiveness Analysis Registry of the Tufts Medical Center [25]. We assumed that individuals who were treated and cured from the disease would have the same utility weight as the general Ethiopian population (0.94) [24], which is in agreement with the previous study which showed no major difference in utility weights between those population groups [26].

**Table 1:** Model parameters point estimate values and ranges

Parameter			Point estimate	Plausible Range	Distribution	Reference
***Treatment efficacy (success rate, %)***						
	Fluconazole		63.0	57.0–74.0	Beta	Mills et al., 2009 [21]
	Caspofungin		76.1	63.6–78.0	Beta	Mills et al., 2009 [21]
	L-AmB		72.9	66.4–76.0	Beta	Mills et al., 2009 [21]
***Mortality rate (%)***						
	Fluconazole		28.4	16.6–31.4	Beta	Mills et al., 2009 [21]
	Caspofungin		33.8	26.4–34.2	Beta	Mills et al., 2009 [21]
***IC/C recurrence rate (%)***			4.4	1.4–13.0	Beta	Ásmundsdóttir et al., 2012; Muñoz et al., 2016; Reboli et al.,2007; Pappas et al., 2007 [20, 22, 27, 28]
***Utilities***						
	Patient with IC/C		0.72	0.50–0.94	Beta	CEA Registry, Tufts Medical Center [25]
	Healthy or survivors		0.94	0.84–0.94	Beta	Granja et al., 2004; Welie et al., 2020 [24, 26]
***Costs (US$)***						
	***Loading dose cost***					
		Fluconazole 800 mg per day	$3	$2–5	Gamma	TASH and MCM
		Caspofungin 70 mg per day	$179	$150–200	Gamma	MCM
		L-AmB (3 mg/kg per day for an average weight of 70 kg)	$198	$190–215	Gamma	TASH and MCM
	***Maintenance dose***					
		Fluconazole 400 mg per day	$17	$15–30	Gamma	TASH and MCM
		Caspofungin 50 mg per day	$2322	$2000–2500	Gamma	MCM
		L-AmB (3 mg/kg per day for an average weight of 70 kg)	$2574	$500–2,700	Gamma	TASH and MCM
	Hospitalization cost per day		$5	$1–35	Gamma	TASH and MCM
	Diagnostic and monitoring costs		$76	$50–100	Gamma	TASH and MCM

TASH: Tikur Anbesa Specialized Hospital; MCM: Myung Sung Christian Medical General Hospital

#### Cost

All cost data were obtained from Tikur Anbessa Specialized Hospital (TASH) and/or Myung Sung Christian Medical General Hospital (MCM) records in Addis Ababa, Ethiopia. The mean total medication costs per patient were U$20 for fluconazole (U$3 for the loading dose and U$17 for the maintenance dose), U$2,501 for caspofungin (U$179 for loading dose and U$2,322 for maintenance dose), and U$2,772 for L-AmB. We estimated hospitalization cost per day of U$5 and the average cost of diagnosis and monitoring tests (such as chest X-ray, computed tomography scan, complete blood count, renal function test, liver function test, electrolyte test) was U$76 per patient. All costs are expressed in 2021 US$ (1US$=43.3 Ethiopian Birr) [29].

### Ethics

This study was approved by the Institutional Ethics Review Board of the School of Pharmacy, College of Health Sciences, Addis Ababa University, Ethiopia. The Ethics Review Board of the School of Pharmacy, Addis Ababa University, Ethiopia waived the requirement for written informed consent since cost data were obtained from historical records, in accordance with national and institutional guidelines. Permission was also obtained from the study hospitals to collect cost data. All methods were carried out in accordance with relevant guidelines and regulations.

### Analysis

In the base-case analysis, we consider hypothetical IC/C patients aged 39 years (based on the mean age of adult inpatients at TASH.

We performed deterministic and probabilistic sensitivity analyses to assess the impact of parameter uncertainties and the robustness of our analysis. In the deterministic sensitivity analysis, we assessed parameter value uncertainty by varying each input variable within a plausible range of values presented in Table 1. The plausible cost range were estimated using the lowest and highest costs of each service in the study hospitals, while the probability and utility ranges were obtained from other sources. We also perform probabilistic sensitivity analysis, running 10,000 Monte Carlo simulations, in which all input variables were allowed to vary simultaneously according to the predefined probability distribution (i.e., gamma distributions for costs, and beta distributions for probabilities and utilities).

## Results

### Base-case analysis

The discounted and undiscounted life years, QALYs, costs, and ICURs are presented in Table 2. Our base-case analysis showed that caspofungin-initiated treatment of IC/C was both more effective and more expensive than fluconazole-initiated treatment. Caspofungin-initiated treatment followed by L-AmB as second-line treatment was associated with an expected 12.86 QALYs and a cost of US$7,714 (discounted). Fluconazole-initiated therapy with caspofungin used as second-line treatment resulted in an expected 12.30 QALYs and a cost of US$3,217 (discounted), while fluconazole-initiated therapy followed by L-AmB produced 10.92 QALYs and a cost of US$2,781(discounted).

**Table 2:** Base-case analysis results for caspofungin- versus fluconazole-initiated treatment strategies for hospitalized Ethiopian patients with IC/C

		Treatment Strategies			Incremental		
		**CASPO** *->* **L-AmB** [1]	**FLU** *->* **CASPO** *->* **L-AmB** [2]	**FLU** *->* **L-AmB** [3]	[1] vs. [2]	[2] vs. [3]	[1] vs. [3]
**Discounted at 3%**							
	Life years	13.81	13.21	11.73	0.60	1.48	2.08
	QALY	12.86	12.30	10.92	0.56	1.38	1.94
	Cost (US$ 2021)	7714	3217	2781	4497	436	4933
	ICUR (US$/QALY)	-	-	-	**8030**	**316**	**2543**
**Undiscounted (0%)**							
	Life years	20.69	19.67	17.18	1.02	2.49	3.51
	QALY	19.25	18.31	15.99	0.94	2.33	3.26
	Cost (US$ 2021)	10,748	3329	2875	7,419	454	7873
	ICUR (US$/QALY)	-	-	-	**7893**	**196**	**2415**

CASPO: Caspofungin; FLU: Fluconazole; L-AmB: Liposomal amphotericin B

The use of caspofungin as first-line treatment for IC/C was not cost-effective when compared to fluconazole-initiated therapy. Compared to fluconazole-initiated therapy with caspofungin as second-line therapy, the caspofungin-initiated treatment resulted in an incremental 0.56 QALYs gained (0.94 QALYs undiscounted) at an incremental cost of US$4,497 (US$7,419 undiscounted); translating to US$ 8,079/QALY. We also compared caspofungin-initiated therapy to fluconazole-initiated treatment followed by L-AmB, and found that caspofungin-initiated therapy was more effective and more expensive, yielding an ICUR of U$2,545/QALY. In the base-case analysis, fluconazole-initiated treatment with caspofungin as second-line treatment was cost-effective compared to fluconazole-initiated treatment followed by L-AmB with an ICUR of US$316/QALY.

### Sensitivity analysis

#### Deterministic sensitivity analysis

**Fig. 2:**
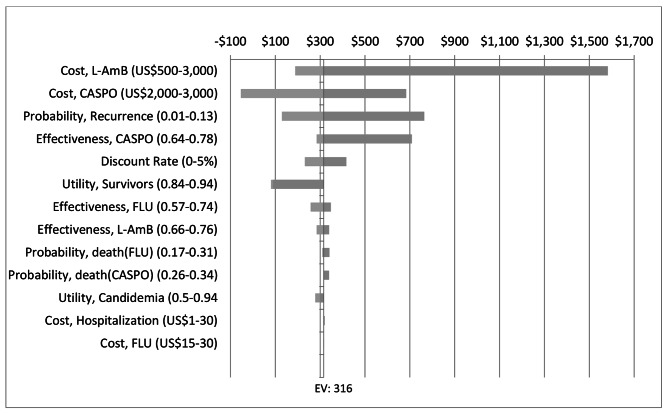
Deterministic sensitivity analysis result of fluconazole-initiated treatment followed by caspofungin versus fluconazole-initiated followed by L-AmB. CASPO: Caspofungin; FLU: Fluconazole; L-AmB: Liposomal amphotericin B

**Fig. 3:**
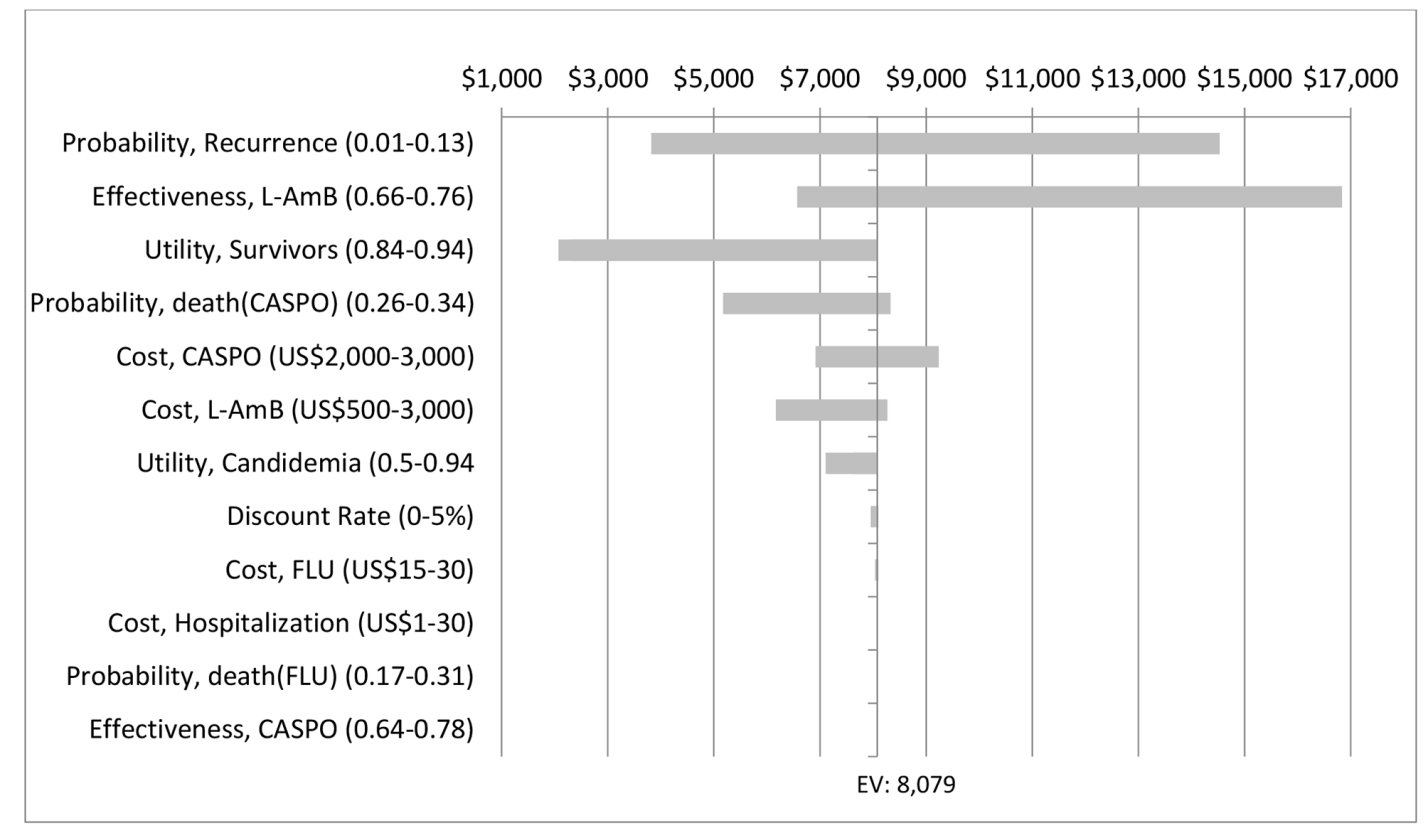
Deterministic sensitivity analysis results of caspofungin-initiated treatment versus fluconazole-initiated treatment with caspofungin as second-line treatment. CASPO: Caspofungin; FLU: Fluconazole; L-AmB: Liposomal amphotericin B

The results of the deterministic sensitivity analysis are presented in Figs. 2 and 3. In the cost-effectiveness analysis of fluconazole-initiated treatment followed by caspofungin versus fluconazole-initiated treatment followed by L-AmB strategies, a tornado diagram showed that the cost-effectiveness was most sensitive to the cost of medications (L-AmB and caspofungin), probability of infection recurrence, and effectiveness of caspofungin. Varying these parameters over their plausible ranges resulted in ICUR values exceeding the reference threshold of US$ 476/QALY (i.e., 50% of Ethiopia’s GDP/capita) (Fig. 2).

Further, we found that the probability of infection recurrence, L-AmB treatment efficacy, and mortality from infection during caspofungin therapy were the most influential variables when comparing caspofungin-initiated therapy to fluconazole-initiated treatment with caspofungin as second-line treatment (Fig. 3). Changing these parameters values over their plausible range, however, did not result in ICUR values below the cost-effectiveness threshold.

#### Probabilistic sensitivity analysis

**Fig. 4:**
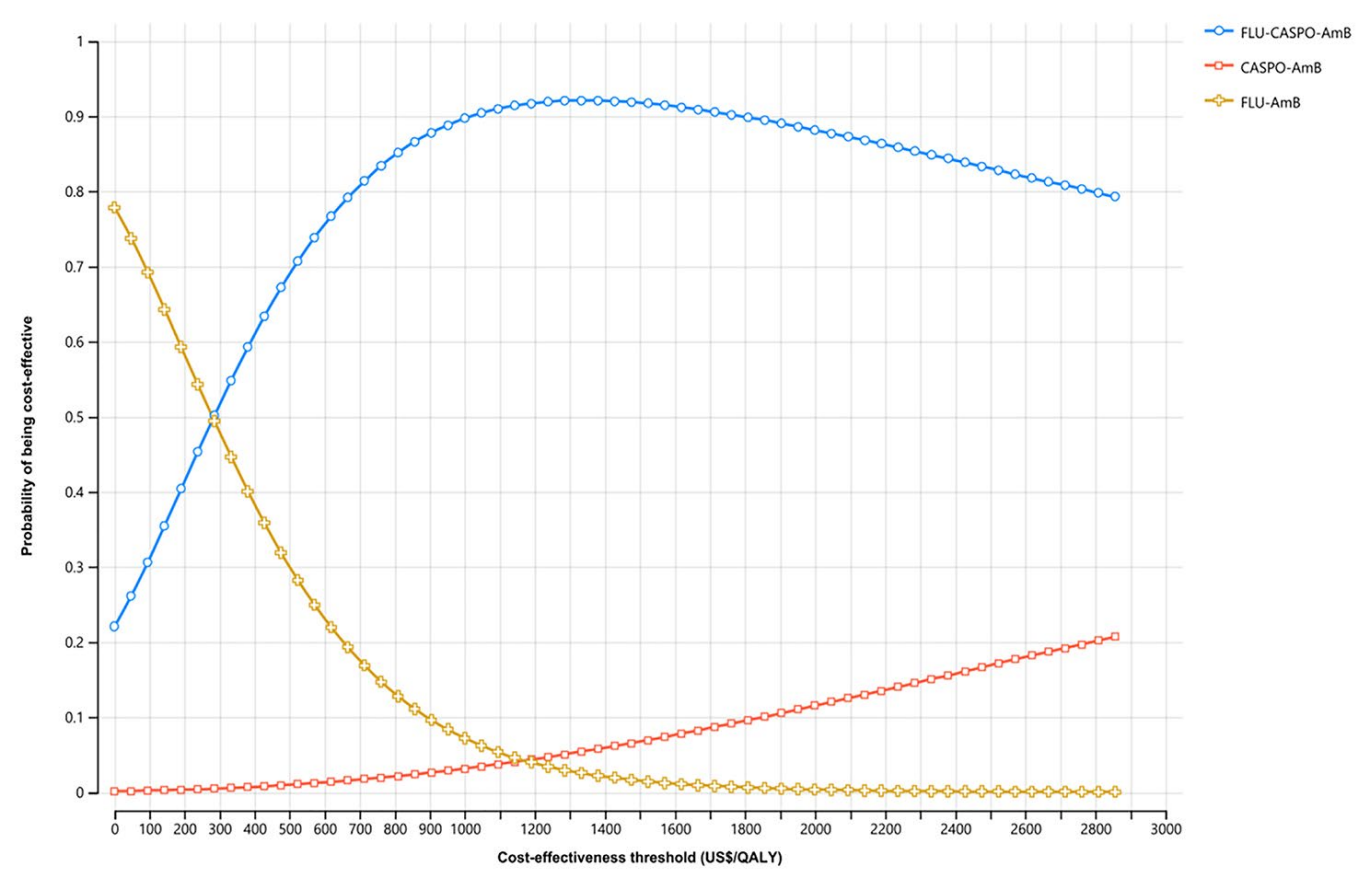
Cost-effectiveness acceptability curves

The cost-effectiveness acceptability curve in Fig. 4 shows the proportion of simulations where each treatment alternative was cost-effective at various cost-effectiveness thresholds. At a cost-effectiveness threshold of US$ 476/QALY (i.e. 50% of Ethiopian GDP/capita), our probabilistic sensitivity analysis showed that fluconazole-initiated treatment followed by caspofungin was more likely to be cost-effective in 67.2% simulations. At cost-effectiveness thresholds of US$952 (i.e., 1xGDP/capita) and US$ 2,856/QALY (i.e., 3xGDP/capita), the probability of fluconazole-initiated treatment followed by caspofungin being cost-effective was 88.8% and 79.3%, respectively. The probability of caspofungin-initiated treatment being cost-effective was 0.9% at a cost-effectiveness threshold of US$476 and 20.7% at threshold US$ 2,856/QALY. Caspofungin-initiated therapy was more likely to be cost-effective when the cost-effectiveness criteria was > 8 times Ethiopian GDP/capita.

## Discussion

Invasive candidiasis is associated with high morbidity and mortality in Ethiopia, though the exact incidence is unknown due to lack of comprehensive epidemiological data. Despite the growing burden of the disease, patient care remains challenging owing to a lack of diagnostic resources and context-specific treatment protocols, as well as drug shortages [6]. Our study compared the cost-effectiveness of caspofungin and fluconazole-initiated therapies for the treatment of hospitalized patients with IC/C. Caspofungin-initiated treatment was not cost-effective when compared to fluconazole-initiated treatments in Ethiopia, which is consistent with studies from other (high resource setting) countries [30, 31]. Ou et al.’s study based on Taiwan’s National Health Insurance [31] indicated that caspofungin was dominated compared to fluconazole, resulting in an incremental cost of US$ 4,983 and an expected 0.49 life years lost. Garu et al.’s study from Spain’s National Health System perspective [30] showed that as compared to fluconazole, caspofungin was associated with an incremental cost-effectiveness ratio of €27,339 per successful treatment, which was below the Spanish cost-effectiveness threshold, suggesting that caspofungin is a cost-effective agent. In our sensitivity analysis, the results remain stable, confirming the robustness of our findings.

Although caspofungin is more effective than fluconazole, it has also been associated with high drug acquisition costs. The use of caspofungin as a second-line drug to fluconazole, therefore, could be a viable alternative for Ethiopia and other low-resource setting countries, given their limited budgets. We could not identify any cost-effectiveness studies that assessed the use of caspofungin as a second-line alternative. Our study compared fluconazole-initiated treatment alternatives and showed that fluconazole-initiated treatment followed by caspofungin was cost-effective compared to fluconazole-initiated treatment followed by L-AmB. Therefore, if a targeted therapeutic decision cannot be made due to a lack of microbiological data, our findings support the use of caspofungin as a second-line treatment option for hospitalized IC/C patients.

While our study utilized a robust model with relevant sensitivity analyses, it has some limitations. Our analysis is for empiric treatment in the absence of microbiology workup and targeted therapies. It is worth noting that fluconazole, unlike caspofungin, has high resistance rates and is ineffective against *Candida* biofilm. We recommend that hospital microbiology departments improve their ability to isolate fungi and test susceptibility to antifungal medications in order to guide targeted therapies [10, 32]. Due to lack of local data, some of our input parameter values such as health state utilities, disease incidence, and associated mortality were obtained from the literature, which might not be applicable to the Ethiopian population. Our findings, however, remained stable in sensitivity analyses, confirming the robustness of our findings. Nonetheless, local effectiveness studies of antifungal treatments and other health-related outcome data such as health state utilities are important to improve context-relevance for future cost-effectiveness analyses. Despite these shortcomings, to the best of our knowledge, this is the first cost-effectiveness study of caspofungin and fluconazole for primary treatment of IC/C in Ethiopia. The findings of the study can inform Ethiopian guidance on antifungal agents for empiric treatment for hospitalized patients with IC/C.

## Conclusion

Our study showed that using caspofungin as first-line treatment for hospitalized patients with IC/C was not cost-effective when compared to fluconazole-initiated treatment that includes either caspofungin or L-AmB as second-line treatment. Fluconazole as a first-line treatment followed by caspofungin was found to be cost-effective compared to fluconazole-initiated treatment followed by L-AmB therapies.

## Data Availability

No additional data are available for this analysis; however, any model-related requests should be directed to the corresponding author.

## List of abbreviations

CASPO: Caspofungin
FLU: Fluconazole
GDP: Gross domestic product
IC/C: Invasive candidiasis or candidemia
ICUR: Incremental cost-effectiveness ratio
LMICs: Low- and middle-income countries
LYs: Life years
L-AmB: Liposomal amphotericin
QALY: Quality-adjusted life years
WHO: World Health Organization
